# Non Invasive Follicular Thyroid Neoplasm with Papillary like nuclear features (NIFTP), A time for change in Pakistan

**DOI:** 10.12669/pjms.36.2.1123

**Published:** 2020

**Authors:** Sumerah Jabeen, Saira Fatima, Aisha Sheikh, Najmul Islam

**Affiliations:** 1 Dr. Sumerah Jabeen, FCPS. Endocrine Fellow, The Aga Khan University Hospital, Karachi, Pakistan; 2 Dr. Saira Fatima, FCPS. Consultant, The Aga Khan University Hospital, Karachi, Pakistan; 3 Dr. Aisha Sheikh, FCPS. Consultant, The Aga Khan University Hospital, Karachi, Pakistan; 4 Prof. Najmul Islam, FRCP. Consultant, The Aga Khan University Hospital, Karachi, Pakistan

**Keywords:** Noninvasive follicular thyroid neoplasm with papillary like nuclear features (NIFTP), Papillary thyroid cancer, Thyroid tumor

## Abstract

**Objective:**

To determine the cases of Noninvasive Follicular thyroid neoplasm with papillary like nuclear features (NIFTP) in Pakistani population retrospectively. Another objective was to determine their clinical and radiological outcomes with respect to local and systemic disease recurrence, reconfirming the benign course of this new nomenclature in Thyroid tumors by WHO in our population would encourage adopting the new conservative treatment approach in such patients.

**Methods:**

This is a retrospective cohort study conducted at a tertiary care center in Karachi, Pakistan from 2007-2016. All follicular Variant papillary thyroid cancer (FVPTC) reported from a single institute had their histopathology slides reexamined for diagnosing NIFTP as per the new WHO criteria. These cases were then followed retrospectively from their diagnosis onset through their medical and electronic health record for any local or systemic disease recurrence.

**Results:**

There were 199 cases of Papillary Thyroid cancer (PTC) which included 22 cases of FVPTC. Eleven cases fulfilled NIFTP criteria with tumor size ranging from 1.1cm to ≥ 5.5cm. All patients in the NIFTP group underwent total thyroidectomy. Nine patients (81.81%) received RAI^131^ therapy. Four (45%) patients had a median follow up of three to four years. There was no disease recurrence seen on both ultrasound and RAI scans of patients in the NIFTP group. Seven patients (87.5%) had normal surveillance thyroglobulin levels except one whereas three patients were lost to follow up. There was no disease recurrence seen both radiologically and biochemically in the NIFTP group.

**Conclusion:**

Our study favors the low risk nature of NIFTP with no disease recurrence in the cases studied and encourages de-escalation of treatment.

## INTRODUCTION

There has been an increased incidence of cancer worldwide and more so in thyroid malignancies owing to intensified surveillance and early detection of cancers with relatively indolent behavior, phenomena commonly referred to as “cancer over diagnosis”.^1^,^2^ According to Globocan statistics in 2012, 48% of all new cases of thyroid cancer were diagnosed in Asia.^3^

Follicular variant papillary thyroid carcinoma (FVPTC) is the most common variant of papillary thyroid carcinoma (PTC), constituting 9% to 22.55% of PTC.^4^ It is further subdivided into noninvasive which includes encapsulated/well demarcated and infiltrative follicular variants.

In 2016 the Endocrine pathology society proposed a new name for the cases diagnosed as EFVPTC as “Noninvasive follicular thyroid neoplasm with papillary like nuclear features (NIFTP)”, eliminating the word “cancer” due to its more indolent nature.^5^ They proposed conservative treatment of this relatively benign condition, stating lobectomy alone without the adjuvant RAI is adequate for ensuring the long term recurrence free outcome in such patients. As per this nomenclature, all tumors previously diagnosed as noninvasive FVPTC would qualify as NIFTP.^6^ This new nomenclature has now been endorsed by WHO.^7^ However NIFTP is not well validated in oncocytic, sub centimeter, and multifocal lesions.

In Pakistan thyroid cancers constitute 1.2% of malignant tumors.^8^ Currently no data is available in our country regarding NIFTP, therefore we felt a need to look at the NIFTP incidence locally and observe the outcome of such patients with respect to its recurrence which would help us in reinforcing the new conservative approach in such patients.

## METHODS

This is a retrospective cohort study conducted at a tertiary care center in Karachi, Pakistan from 2007-2016. This study was approved (Ref. No. 4782-Med-ERC-17 dated June 1, 2017) by the Ethical Review Committee of the hospital.

For the purpose of this study the data for all the 843 thyroidectomies conducted at our hospital from 2007 to 2016 was taken from the Health Information Management Systems (HIMS) department. Clinical data was collected through a questionnaire filled after going through patient’s medical files whereas laboratory data and radiological investigations were collected through the electronic health record of the hospital. We analyzed patient demographics including age at the diagnosis and gender. Tumor size was recorded as per the histopatholgy and clinical outcomes were defined as either recurrence of the disease, no recurrence or lost to follow up. These outcomes were determined by following patients with surveillance ultrasound, whole body scan along with serum thyroglobulin levels with different cut offs for both TSH stimulated (≤ 1ng/ml) and non stimulated thyroglobulin levels (≤ 0.2 ng/ml) as per American Thyroid Association (ATA) and thyroglobulin antibody levels (<20 IU/ml).

The histopathology reports were evaluated for 843 thyroidectomies done from 1^st^ January 2007 to 31^st^ December 2016 after which 22 cases of FVPTC were filtered. The histopathology slides of all the FVPTC were reviewed by a single histopathologist as per the WHO criteria proposed for NIFTP (14), including encapsulated thick, thin or partial capsule with clear demarcation from adjacent tissue, follicular growth pattern with <1% papillae, no psammoma bodies, <30% solid/trabecular/insular growth pattern with a nuclear score between 2-3 and no lymphovascular or capsular invasion, no tumor necrosis and less than 3 mitoses per 10 HP. (Table-I)

**Table-I:** Comparison of characteristics between NIFTP and FVPTC.

Characteristics	NIFTP	FVPTC
Capsule	Encapsulated/circumscribed	Encapsulated with capsular or vascular invasion
Follicular Growth Pattern with;		
a)Papillae	<1%	>1%
b)Psammoma bodies	Absent	May be present
c)Growth Pattern	<30% solid, trabecular, or insular	≥30% solid, trabecular, or insular
Nuclear Features Score	2-3	Usually 3
Lymphovascular Invasion	No	May be present
Capsular Invasion	No	May be present
Tumor necrosis	Yes No	No
Mitotic Activity	<3 /10 hpf	≥3/10HPF

On the basis of this reexamination of slides, cases were divided into two groups, NIFTP and FVPTC respectively. <1 cm micro carcinomas, multifocal tumors were excluded from the study.

### Plan of Analysis

Mean with standard deviation was reported for symmetrically distributed continuous variable while median with interquartile range was reported for asymmetrically distributed continuous variable. Normality of the continuous variable was assessed through histogram by plotting a normal density plot on the graph. Frequencies with percentages were reported for all categorical variables. All analyses were performed on Stata version 12.

## RESULTS

Initial screening of 199 cases of PTC from Jan 1, 2007 to Dec 31, 2016 revealed 22 cases of FVPTC in which 11 met the strict inclusion criteria of NIFTP, 11 were classified as FVPTC. This translates into an incidence of 5.5% of NIFTP cases at our institution within the study period. When comparing 11 cases of NIFTP and 11 cases of FVPTC (as evident from Table-II), a head to head comparison revealed that the median age of the study participants in the NIFTP group was 33 years while it was 50 years in the FVPTC group. There was a female predominance in both groups, nine (82%) in the NIFTP group versus seven (64%) in the FVPTC group. Six patients underwent total thyroidectomy whereas five initially underwent lobectomy followed by completion thyroidectomy in the NIFTP group whereas in FVPTC group all patients underwent total thyroidectomy. Only one participant in the NIFTP group underwent lymph node dissection as compared to two patients in the FVPTC group. Five (45%) of our study participants falling in the NIFTP group had tumor size of greater than or equal to five centimeters as compared to six (54%) in the FVPTC group. Radioactive therapy was given to nine (81.81%) patients in both the NIFTP and the FVPTC group. Surveillance neck ultrasound and whole body scan revealed no recurrence in the NIFTP group as compared to two study participants having recurrence in the FVPTC group on both ultrasound and RAI surveillance scans. Biochemical surveillance showed seven out of eleven patients in the NIFTP group to have within range thyroglobulin levels whereas only one patient had minimally raised serum thyroglobulin levels (1.3ng/ml), one patient post RAI and two patients following surgery were lost to follow up.

**Table-II:** Comparison of NIFTP (n=11) with FVPTC (n=11) cases – Subgroup Analysis.

S. No.	Variables	No. of study participants in NIFTP group n (%)	No. of study participants in FVPTC group n (%)
1.	Age*	33 (29 – 38)	50 (26 – 65)
2.	Gender	1.Male = 2 (18%)	1.Male = 4 (36%)
		2.Female = 9 (82%)	2.Female = 7 (64%)
3.	Histopathology	NIFTP = 11 (100%)	FVPTC = 11 (100%)
4.	Tumor Size (in max. dimension)	1.1.1-2.9 cm = 2 (18%)	1.1-2.9 cm = 3 (28%)
		2.3-4.9 cm = 4 (37%)	2.3-4.9 cm = 2 (18%)
		3.≥ 5 cm = 5 (45%)	3.≥ 5 cm = 6 (54%)
5.	Patients receiving RAI	1.Yes = 9 (81.81%)	1. Yes = 9 (82%)
		2.No = 01(9.09%)	2. No = 1 (9%)
		3.Lost to follow-up = 1 (9.09%)	3. Lost to follow-up = 1 (9%)
6.	Surveillance neck ultrasound N=8	1.Recurrence = 0 (0%)	1.Recurrence = 2 (25%)
		2.No Recurrence = 8 (100%)	2.No Recurrence = 6 (75%)
		3.Lost to follow-up = 3	3.Lost to follow-up = 3
7.	Surveillance whole body scan N=8	1.Recurrence = 0 (0%)	1.Recurrence = 2 (25%)
		2.No Recurrence = 8 (100%)	2.No Recurrence = 6 (75%)
8.	Surveillance thyroglobulin levels	1.	1.
	a.Stimulated Thyroglobulin levels (ng/ml)	N=1 1.>1 = 0 (0%) 2.≤1 = 1 (100%) 2.	N=2 1.>1 = 1 (50%) 2.≤1 = 1 (50%) 2.
	b.Unstimulated Thyroglobulin levels (ng/ml)	N=7 3.> 0.2 = 1 (14%) 4.≤ 0.2 = 6 (86%)	N=9 3.> 0.2 = 7 (78%) 4.≤ 0.2 = 2 (22%)
9.	Surveillance thyroglobulin antibodies	Positive antibodies (> 20) = 1 (13%)	Positive antibodies (> 20) = 0 (0%)
10.	Follow-up time	1.1-2 years = 1 (11%)	1.1-2 years = 6 (55%)
		2.3-4 years = 4 (45%)	2.3-4 years = 2 (18%)
		3.5-7 years = 3 (33%)	3.5-7 years = 3 (27%)
		4.8-10 years = 1 (11%)	4.8-10 years = 0 (0%)
11.	Outcome with respect to follow-up	N=11	N=11
		1.Recurrence = 0 (0%)	1.Recurrence = 3 (33.33%)
		2.No recurrence (disease-free) = 8 (100%)	2.No recurrence (disease-free) = 6 (66.66%)
		3.Lost to follow-up = 3	3.Lost to follow-up = 2

* Median with interquartile range has been reported since the variable of age is asymmetrically distributed.

Biochemical surveillance in the FVPTC group showed eight out of eleven patients to have raised thyroglobulin levels with disease recurrence noted in four patients. Majority of the participants falling in the NIFTP group had a follow-up time of three to four years, (45%) as compared to those falling in the FVPTC group who had a median follow-up time of one to two years, (55%). There was no patient having recurrence in the NIFTP group while there were three patients with disease recurrence in the FVPTC group as evidenced by raised Thyroglobulin levels, with two patient showing increased RAI uptake in bones and one showing lymph nodes on U/S imaging.

## DISCUSSION

The encapsulated noninvasive follicular variant papillary thyroid carcinoma was revised in nomenclature as NITFP owing to its indolent behavior. The molecular analysis demonstrates that these lesions are driven by clonal genetic alterations and therefore should be more appropriately classified as neoplasms.^9^ Till recently these tumors have been treated like conventional thyroid cancers with treatment including total thyroidectomy, I^131^ RAI ablation and thyroid hormone suppression treatment with levothyroxine.^10^

Our data was collected from a single tertiary care center care in Pakistan over a period of 10 years’ duration. There were 199 cases of PTC including 22 cases reported as FVPTC, out these 22 cases 11 cases met the criteria of NIFTP whereas the remaining 11 were classified as FVPTC other than NIFTP. The smallest tumor size noted in the NIFTP group was 2.0 x 1.5 x 1.0 cm whereas the largest NIFTP noted was 6.6 x 4.9cm. Only one patient in the NIFTP group had his lymph node dissection done along with total thyroidectomy as the patient had concomitant laryngeal carcinoma therefore this lymph node dissection was done for staging workup of the primary cancer. Only one participant in the NIFTP group had an unstimulated thyroglobulin level of 1.35ng/ml (Normal range <0.2) but there has been no recurrence noted after two years of follow up. This study showed an overall incidence of NIFTP as 5.5% of all PTC. An Indian study published recently found an incidence of 9.4% (33 cases of NIFTP in 349 cases of PTC) retrospectively.^11^ Bychkov et al. on the other hand presented data from East Asia, recorded only 1.5% (range 0-4.7%) which is approximately 10 times less compared to western series in which NIFTP was reported as 16-23% of all PTC in North America and European populations.^12^,^13^

Hypothetically this striking difference could be due to various reasons including geographic and ethnic differences among Asian populations, the method of nodule detection, as in our country it is usually detected on palpation whereas in USA and many other countries nodules detected as an incidental finding on imaging are also examined. The pathological interpretation remains another important aspect of this difference. This difference was also taken into account by Nikifirov paper where inter observer agreement requiring to label a specimen as NIFTP was set at only 50%.

All NIFTP diagnosed retrospectively in our study showed no disease recurrence compared to FVPTC group where four patients had disease recurrence despite similar aggressive treatment as given in the NIFTP group thus reconfirming the indolent nature of NIFTP. Sheethla et al. describes the safest figure of recurrences/metastasis with NIFTP cases to be 0.5%.^14^

### Limitation of the study

Our study has some limitations due to the retrospective nature of the data collection, being a single center study with smaller cohort, aggressive treatment given to all NIFTP except one patient and few lost to follow up cases. Also currently we do not have genetic mutation testing available in our country.

## CONCLUSION

Our study although a single institute based study with limited number of patients favors NIFTP as a low risk tumor with rare incidence of adverse outcomes. It favors de-escalation of clinical management of NIFTP as such patients are unlikely to benefit from completion thyroidectomy and RAI therapy. Further studies are required to validate our study in Pakistan and also a further follow up of this data would be important for reconfirming the benign outcome. In the long run it can have beneficial effects of reducing the financial burden of this overtreatment and eliminating the psychological impact of the cancer diagnosis in such patients. We also recommend dissemination of knowledge regarding diagnostic criteria of NIFTP to health care providers involved in thyroid cancer management.

### Author`s Contribution:

**SJ, SF** conceived and designed study, analyzed data with histopatholgy slides reexamined and manuscript writing.

**AS,** contributed in literature search, data collection and drafting final manuscript.

**NI** supervised the study and was the critical reviewer.
